# Hierarchical modelling of variance components makes analysis of resolvable incomplete block designs more efficient

**DOI:** 10.1007/s00122-024-04639-4

**Published:** 2024-05-16

**Authors:** Marcin Studnicki, Hans Peter Piepho

**Affiliations:** 1 Department of Biometry, Institute of Agriculture, Warsaw, University of Life Sciences, Nowoursynowska 159, 02-776 Warsaw, Poland; 2 Biostatistics Unit, Institute of Crop Science, University of Hohenheim, Fruwirthstraße 23, 70599 Stuttgart, Germany

## Abstract

The standard approach to variance component estimation in linear mixed models for alpha designs is the residual maximum likelihood (REML) method. One drawback of the REML method in the context of incomplete block designs is that the block variance may be estimated as zero, which can compromise the recovery of inter-block information and hence reduce the accuracy of treatment effects estimation. Due to the development of statistical and computational methods, there is an increasing interest in adopting hierarchical approaches to analysis. In order to increase the precision of the analysis of individual trials laid out as alpha designs, we here make a proposal to create an objectively informed prior distribution for variance components for replicates, blocks and plots, based on the results of previous (historical) trials. We propose different modelling approaches for the prior distributions and evaluate the effectiveness of the hierarchical approach compared to the REML method, which is classically used for analysing individual trials in two-stage approaches for multi-environment trials.

**Supplementary Information:**

The online version contains supplementary material available at 10.1007/s00122-024-04639-4.

## Introduction

The yield of modern cultivars (especially for cereals), while having increased substantially over the past decades, often displays diminishing genetic variance, meaning that differences between the best and worst cultivars may be small (Laidig et al. 2017). Thus, in order to reliably evaluate and compare cultivars, it becomes ever more important to accurately estimate the cultivar means and their differences. In many countries in Europe and in the world, such evaluation of cultivars is carried out in official registration or post-registration multi-environment trials (MET), providing the basis for cultivar recommendations (Welham et al. 2010; van Eeuwijk et al. 2016).

Statistical analysis of METs by linear mixed models (LMM) is usually performed with the residual maximum likelihood (REML) method, which was, in fact, originally proposed for the specific purpose of recovery of inter-block information in the paper by Patterson and Thompson (1971). The REML methods are based on maximising the likelihood of the data for a specific set of error contrasts and are equivalent to the analysis of variance method for estimating variance components (Nelder 1965a) when designs are balanced, and all REML estimates are positive. LMM allow flexible assumptions about the variance–covariance matrix for random and residuals effects (So and Edwards 2009; Hu and Spilke 2011; Schielzeth et al. 2020). Analysis of MET can be carried out using two approaches—single-stage and two-stage approaches. A single-stage approach involves using a model for the plot data from all environments of the MET with all experimental design and treatment effects. However, when MET data are extensive, using a single-stage approach can be computationally demanding (Buntaran et al. 2019). In day-to-day operations, therefore, researchers are more likely to use a two-stage approach, in which individual trials are analysed in the first stage and only in the second stage is a combined analysis performed based on the treatment means per trial obtained in the first stage. With proper weighting, such a stage-wise approach is essentially equivalent to a single-stage approach (Piepho et al. 2012; Damesa et al. 2017). In two-stage analyses, it is important that the estimates of the variance components obtained in the first stage have as much accuracy as possible. Not only is the accurate estimation of variance components from individual trials important for effective evaluation in the second stage of the analysis. Many researchers and breeders are also interested in effective analysis of individual trials per se, because they need to make a reliable assessment of genotypes and varieties in individual experiments, e.g. to assess genotype–environment interaction, or because early into the harvest season only a single trial’s data is available for a selection decision. This is a difficult task due to the fact that modern varieties differ very little in terms of functional characteristics, so the importance of very effective estimation of differences between them is increasing (Welham et al. 2010).

One of the most commonly used experimental design for plant breeding trials is the alpha design (Patterson and Williams 1976; Hoefler et al. 2020; John and Williams 1995). This design is resolvable, meaning that is complete replicates, and is subdivided into incomplete blocks. Apart from plot errors, the analysis model comprises design effects for replicates and incomplete blocks, the latter usually being modelled as random for recovery of inter-block information.

It turns out that despite many advantages of the REML method, such as relative simplicity of computation and ease of application of flexible variance patterns, there are drawbacks. One drawback in the context of incomplete block designs is that the variance component for the block effect may be estimated as zero (Sarholz and Piepho 2008), which can compromise the recovery of inter-block information and hence reduce the accuracy of Best Linear Unbiased Predictions (BLUPs) and Best linear Unbiased Estimators (BLUE) of treatment effects (John 1987; Piepho et al. 2008). This, in turn, may adversely affect the reliability of cultivar evaluation and the credibility of recommendations to farmers.

Due to the development of statistical and computational methods, there is an increasing interest in adopting Bayesian approaches for plant breeding and cultivar evaluation data analysis (Crossa et al. 2011; Jarquín et al. 2016). In particular, a lot of studies were done on the development of Bayesian approaches for the additive main effects and multiplicative interaction (AMMI) model (Josse et al. 2014; Bernardo Júnior et al. 2018; Romão et al. 2019). The AMMI model is one of the tools used to assess genotypic (cultivar) stability and adaptability patterns. Bayesian approaches have been implemented for the AMMI model with both homogeneous residual variance (Viele and Srinivasan 2000; Teodoro et al. 2019) and heterogeneous residual variance (da Silva et al. 2015, 2019). Bayesian approaches have also been adapted in other approaches to study genotype × environment interaction such as the genotype plus genotype × environment (GGE) model (de Oliveira et al. 2016) and for factor analytic (FA) models (de los Campos and Gianola 2007; Dunson 2008; Nuvunga et al. 2019). While there is quite a lot of work on Bayesian methods for AMMI and GGE models, with a focus on between-trial effects, relatively little work has been done on simpler random-effects models as needed for the randomisation-based analysis of individual trials (Theobald et al. 2002; So and Edwards 2009; Przystalski and Lenartowicz 2020). This is surprising because objective prior information on within-trial components of variance is abundantly available in plant breeding programmes and variety testing systems.

Various estimation methods can be used in the application of Bayesian approaches. One of the more popular options is to use algorithms for sampling from a probability distribution, such as the Markov Chain Monte Carlo (MCMC) method with Gibbs sampling to approximate the posterior distribution in case of conjugate priors. These methods are popular, e.g. in ecological research (King et al. 2009; Hooten and Hobbs 2015; Dorazio 2016) and animal genetics (Rasch and Mašata 2011; Villemereuil 2019).

One of the advantages of the Bayesian approach is the need to define a prior probability distribution for parameters of interest. This allows taking into account the knowledge about parameters available before a new experiment using a hierarchical model. A prior distribution can be based on information available for the cultivars, its pedigree and trial locations (da Silva et al. 2019). The incorporation of this information into the model allows improving the analysis of new experiments. However, quite surprisingly, the use of such additional information in cultivar evaluation and MET analysis is as yet very rare. One example is found in Theobald et al. (2002). Here, we focus on the within-trial variance components, because databases on the past trials usually provide a solid basis to objectively inform priors. This great potential so far has remained largely untapped. The main purpose of our paper is to open a path to exploit this dormant information. Increasing the accuracy of the assessment of variance components can be achieved by applying a hierarchical model making use of existing experimental results. Such an approach also can avoid zero estimates of variance components for incomplete blocks, thus potentially improving the recovery of inter-block information. In order to increase the precision of the analysis of individual trials laid out as alpha designs, we here make a proposal to create a prior distribution for variance components of replicates, blocks and plots, based on the results of previous (historical) individual trials. We consider different assumptions for the prior distributions for variance components and using simulation evaluate the effectiveness of two hierarchical modelling approaches to the REML method classically used with to analysis individual trials from METs. Our hierarchical models are implemented using both an Empirical Bayes and a fully Bayesian approach.

## Material and methods

### Historical data set

**Fig. 1:**
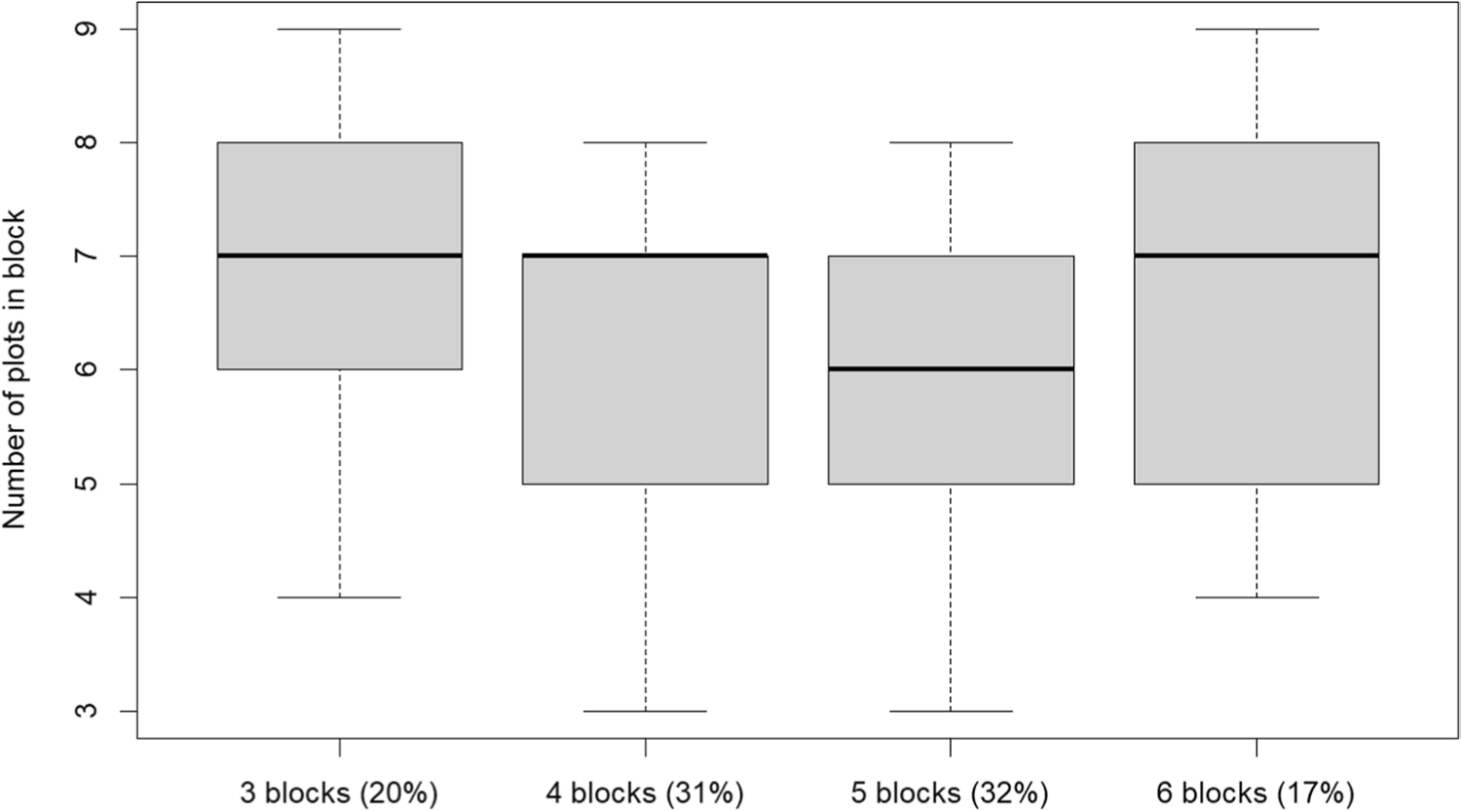
Number of plots in block across historical data set. The percentage of individual trials with the given number of blocks per replicate in study data set is shown in brackets

The historical data set we use constitutes a collection of individual trials with wheat from the Polish Post-Registration Variety Testing System (PVTS), an official cultivar testing system used for informing recommendations to farmers. We used data from individual trials carried out between the 2009/2010 and 2018/2019 growing seasons. The number of trial locations varied from 35 in 2017/2018 to 43 in 2011/2012. The number of varieties ranged from 51 in 2009/2010 to 63 in 2018/2019. The total number of individual trials was equal to 387. The PVTS experiments were laid out as alpha designs with two replicates (complete blocks), with the number of blocks (incomplete blocks) per replicate depending on the number of cultivars in the individual trials, ranging from 3 to 6. Figure 1 presents the dependence of the number of plots per block on the number of blocks in the studied trials, varying from 3 to 9. Routinely, results from individual trials are analysed using the REML method. The historical data set is used here to inform the priors of the hierarchical modelling approaches used to analyse the individual trials.

### Models

For individual trials laid out as alpha designs, we assume the linear mixed model

1 $$ y_{ijl} = \mu + \tau_{i} + \gamma_{j} + \rho_{l\left( j \right)} + \varepsilon_{ijl} $$

where $y_{ijl}$ is the yield response of the *i*th cultivar in the *l*th block within the *j*th replicate, $\mu$ is the overall intercept, $\tau_{i}$ is the fixed effect of the *i*th cultivar, $\gamma_{j}$ is the random effect of the *j*th replicate, $\rho_{l\left( j \right)}$ is the random effect of the *l*th block nested in the *j*th replication and ε_*ijl*_ is the error effect.

The standard method for estimating variance components is REML. When the number of blocks or replicates is small, it is quite likely that variance component estimates by REML are zero. Thus, using REML estimates directly to build a prior for variance components is problematic, as it would require modelling a spike at zero. To circumvent this problem, our hierarchical modelling approach is based on sums of squares of a sequential analysis of variance (ANOVA) for model (1) computed for the individual trials (Sarholz and Piepho 2008). This two-stage approach also has the advantage of greatly reducing the computational burden compared to a single-stage approach based on a model for the individual plot data specified for all trials simultaneously, which may be many. Our approach can be regarded as a hierarchical extension of classical ANOVA estimation of variance components (Searle et al. 1992).

Specifically, from an ANOVA for an individual trial with sequential sums of squares (Type I SS in SAS), in which treatments are fitted before all design effects, we obtain the sums of squares for replicates, SS_*r*_, blocks, SS_*b*_, and error, SS_*e*_, as well as the corresponding expected mean squares, which are functions of the three variance components for replicates, blocks and error (Table 1). We compute the sums of squares and the expected mean squares using the MIXED procedure in SAS. Classical ANOVA estimation of variance components equates observed mean squares to their expected values and solves the resulting set of equations for the variance components (Searle et al. 1992). This is not done here. Instead, we use the expected mean squares in Table 1, which are linear functions of the unknown variance components, to formulate a hierarchical model for the variance components that use the observed sums of squares as the response (Sarholz and Piepho 2008), as is described next.

**Table 1:** Sequential ANOVA table for random design effects based on linear mixed model for an alpha design (see main text)

Source	Degrees of freedom (d.f.)	Sum of squares (SS)	Mean square (MS)	Expected mean squares E(MS)
Replicates	*v*_*r*_	SS_*r*_	${\text{MS}}_{r} = {{{\text{SS}}_{r} } \mathord{\left/ {\vphantom {{{\text{SS}}_{r} } {v_{r} }}} \right. \kern-0pt} {v_{r} }}$	$\sigma_{e}^{2} + c_{1} \sigma_{b}^{2} + c_{2} \sigma_{r}^{2}$
Blocks	*v*_*b*_	SS_*b*_	${\text{MS}}_{b} = {{{\text{SS}}_{b} } \mathord{\left/ {\vphantom {{{\text{SS}}_{b} } {v_{b} }}} \right. \kern-0pt} {v_{b} }}$	$\sigma_{e}^{2} + c_{3} \sigma_{b}^{2}$
Error	*v*_*e*_	SS_*e*_	${\text{MS}}_{e} = {{{\text{SS}}_{e} } \mathord{\left/ {\vphantom {{{\text{SS}}_{e} } {v_{e} }}} \right. \kern-0pt} {v_{e} }}$	$\sigma_{e}^{2}$

Treatments, not shown in the sequence, are fitted before design effects. c_1_, c_2_ and c_3_ are known constants depending on the design computed according to Goodnight and Speed (1978)

Conditionally on the variance components, $\sigma = \left( {\sigma_{r}^{2} ,\sigma_{b}^{2} ,\sigma_{e}^{2} } \right)^{T}$, the sums of squares have independent scaled central Chi-squared distributions (see Appendix), i.e.

$$ \frac{{{\text{SS}}_{i} |\sigma }}{{E\left( {{\text{MS}}_{i} } \right)}}\sim \chi_{{v_{i} }}^{2} \left( {i = r,b,e} \right) $$

For the variance component vector $\sigma$, we assume four different parameterisations, which for our hierarchical approach simultaneously constitutes different prior distribution specifications. The first is a trivariate log-normal (Sarholz and Piepho 2008) distribution given by

$$ \left( {\begin{array}{*{20}c} {\log \left( {\sigma_{r}^{2} } \right)} \\ {\log \left( {\sigma_{b}^{2} } \right)} \\ {\log \left( {\sigma_{e}^{2} } \right)} \\ \end{array} } \right)\sim MVN\left[ {\left( {\begin{array}{*{20}c} {\theta_{r} } \\ {\theta_{b} } \\ {\theta_{e} } \\ \end{array} } \right),\left( {\begin{array}{*{20}c} {\varphi_{r}^{2} } & {\varphi_{rb} } & {\varphi_{re} } \\ {} & {\varphi_{b}^{2} } & {\varphi_{be} } \\ {} & {} & {\varphi_{e}^{2} } \\ \end{array} } \right)} \right] $$

For this variance parameterisation, considered as prior distribution in our hierarchical approach, we have two sets of hyper-parameters, i.e. the means vector $$\theta = \left[ {\begin{array}{*{20}c} {\theta_{r} } \\ {\theta_{b} } \\ {\theta_{e} } \\ \end{array} } \right]$$ and the variance–covariance matrix $$\varphi = \left[ {\begin{array}{*{20}c} {\varphi_{r}^{2} } & {\varphi_{rb} } & {\varphi_{re} } \\ {} & {\varphi_{b}^{2} } & {\varphi_{be} } \\ {} & {} & {\varphi_{e}^{2} } \\ \end{array} } \right]$$ of the log-variance components. We fitted this model with two different parameterisations. The first is the one given above. In the second, we ensure that the elements of $\varphi$ are properly constrained using the following parameterisations, where $\lambda_{rr} , \lambda_{bb} , \lambda_{ee} , \lambda_{rb} , \lambda_{re} , \lambda_{be}$ are one-to-one parameterisations of the variance parameters $\varphi_{r}^{2}$, $\varphi_{b}^{2}$, $\varphi_{e}^{2}$, $\varphi_{rb}$, $\varphi_{re}$, $\varphi_{be}$:

$$ \varphi_{r}^{2} = \exp \left( {\lambda_{rr} } \right) $$

$$ \varphi_{b}^{2} = \exp \left( {\lambda_{bb} } \right) $$

$$ \varphi_{e}^{2} = \exp \left( {\lambda_{ee} } \right) $$

$$ \pi_{rb} = \frac{{\exp \left( {2\lambda_{rb} } \right) - 1}}{{\exp \left( {2\lambda_{rb} } \right) + 1}}\begin{array}{*{20}c} , & {\varphi_{rb} = \pi_{rb} \varphi_{r} \varphi_{b} } \\ \end{array} $$

$$ \pi_{re} = \frac{{\exp \left( {2\lambda_{re} } \right) - 1}}{{\exp \left( {2\lambda_{re} } \right) + 1}}\begin{array}{*{20}c} , & {\varphi_{re} = \pi_{re} \varphi_{r} \varphi_{e} } \\ \end{array} $$

$$ \pi_{be} = \frac{{\exp \left( {2\lambda_{be} } \right) - 1}}{{\exp \left( {2\lambda_{be} } \right) + 1}}\begin{array}{*{20}c} , & {\varphi_{be} = \pi_{be} \varphi_{b} \varphi_{e} } \\ \end{array} $$

The exponential specification for the variances makes sure these are positive, whereas the use of the inverse of Fisher’s *z* transformation ensures that a correlation *π* obeys the constraint $\left| \pi \right| < 1$. The inverse of Fisher’s z transformation ensures that correlations will have values in the range between -1 and 1. This second variance prior distribution has the same set of hyper-parameters in the hierarchical approach described further below as the first specification for the trivariate log-normal distribution. The simultaneous use of exponential specification for the variances and the inverse of Fisher’s *z* transformation allows for such a formulated model that will ensure the variance component estimates meet the expected assumption, namely they will be greater than zero.

We further use the Gamma and inverse Gamma distributions as priors for the variances. To fit these, we use the inverse distribution function method [also known as inverse cumulative distribution function (*c*. *d*. *f*.) method] (Piepho and McCulloch 2004; Liu and Yu 2008), which is also used in multivariate simulation (Johnson 1987; p.19). The key idea is that a normal random variable can always be transformed to a standard uniform random variable, which, in turn, can always be transformed to any desired distribution using its inverse *c*. *d*. *f*. Thus, if *Z* is a standard normally distributed random variable, then $U = \Phi^{ - 1} \left( Z \right)$ with $\Phi \left( . \right)$, the inverse *c*. *d*. *f*. of the standard normal, is a uniform [0,1] random variable. Further, $Y = F^{ - 1} \left( U \right)$ with *F* the *c*. *d*. *f*. of an arbitrary distribution. The only limitation of this inverse *c*. *d*. *f*. approach is that it is focussed on the marginal distributions and not on a specific joint distribution. As we require a standard normal random effect as a starting point, we can fix the variances of our trivariate normal distribution at unity and the means at zero, leaving only the correlation as parameters to be estimated. Additional parameters are then needed to model the transformation to the desired marginal distributions for the three variances. Multiplication of the standard Gamma-distributed effect with scale parameter $\beta$ then leads to a random effect with a ${\text{Gamma}}\;\left( {\alpha ,\beta } \right)$ distribution. For inverse Gamma (IG) distribution, we use the fact that if *X* has a ${\text{Gamma}}\;\left( {\alpha ,\beta } \right)$ distribution, then 1/*X* has an IG distribution (Hoff 2009, p.74). For the Gamma and inverse Gamma prior distributions, the shape parameter $\alpha$, the scale parameter $\beta$ and mean vector and variance–covariance matrix of the log-variance components $\varphi$ constitute hyper-parameters.

In the Supplementary Information, we provide the file with SAS code (code_EB_FB.sas) used in our analyses. The applied trivariate log-normal with exponential specification, Gamma and inverse Gamma prior distributions guarantee that the estimated variance components will be positive. The previous studies show that Gamma and inverse Gamma distributions are the most effective in the estimation of variance components (Daniels 1999; Tiao and Tan 1965).

### Specification of the conditional distribution of the sums of squares for given variances

The observed sums of squares (*SS*_*i*_) have a scaled Chi-squared distribution. The Chi-squared distribution is a special case of the Gamma distribution. We use the following parameterisation of the likelihood for a Gamma-distributed random variable:

$$ W\sim {\text{gamma}}\;\left( {\tilde{\alpha },\tilde{\beta }} \right) $$

$$ l\left( {\tilde{\alpha },\tilde{\beta };w} \right) = - \tilde{\alpha }\log \left( {\tilde{\alpha }} \right) - \log \left\{ {\Gamma \left( {\tilde{\alpha }} \right)} \right\} + \left( {\tilde{\alpha } - 1} \right)\log \left( w \right) - w/\tilde{\beta } $$

$$ E\left( W \right) = \tilde{\alpha }\tilde{\beta } $$

$$ {\text{var}} \left( W \right) = \tilde{\alpha }\tilde{\beta }^{2} $$

Here *W* is the Gamma-distributed random variable, and $\tilde{\alpha }$ and $\tilde{\beta }$ are the parameters. For the Chi-squared distribution with *v* degrees of freedom, the first two moments are *v* and 2*v* (Johnson 1987 p.420). Equating moments, we find $\tilde{\beta }$ = 2 and $\tilde{\alpha }$ = *v*/2. In our case, *W* is equal to the scaled Chi-squared random variable. We used the NLMIXED procedure of SAS to fit these models. We cannot use the in-built likelihood for Gamma in the model statement; however, because the Gamma-distributed random variable is a scaled sum of squares, which depends on the variance components, whereas we need the likelihood for the random variable SS_*i*_, which itself is not Gamma-distributed. Thus, we need to write out the above definition as our user-defined likelihood function. Setting

$$ W\left( {{\text{SS}}_{i} } \right) = \frac{{{\text{SS}}_{i} |\sigma }}{{E\left( {{\text{MS}}_{i} } \right)}}\sim \chi_{{v_{i} }}^{2} $$

and observing that this is Gamma-distributed with parameters $\tilde{\beta }$ = 2 and $\tilde{\alpha }$ = *v*_*i*_/2, we may perform a change of random variable in the likelihood (Atkinson 1985 p.86). Thus, if *f*(*W*) denotes the density of *W*, then the conditional density of SS_*i*_, given $\sigma$, is equal to

$$ g\left( {{\text{SS}}_{i} |\sigma } \right) = f\left( {\frac{{{\text{SS}}_{i} |\sigma }}{{E\left( {{\text{MS}}_{i} } \right)}}} \right)\left( {\frac{{\partial W\left( {{\text{SS}}_{i} } \right)}}{{\partial {\text{SS}}_{i} }}} \right) = f\left( {\frac{{{\text{SS}}_{i} |\sigma }}{{E\left( {{\text{MS}}_{i} } \right)}}} \right)\frac{1}{{E\left( {{\text{MS}}_{i} } \right)}} $$

### Empirical Bayes

For fitting the above four prior distributions of the variance components, we used an Empirical Bayesian (EB) approach. To implement the EB approach, we used maximum likelihood (ML) with adaptive Gaussian quadrature to fit our hierarchical model. This approach was implemented using the NLMIXED procedure of SAS, which only allows normally distributed random effects. The Gamma and inverse Gamma distributions can be implemented using the GAMINV function, which computes the inverse cumulative distribution function (*c*. *d*. *f*.) of the standard Gamma distribution with shape parameter $\alpha$.

### Fully Bayesian estimation

The fully Bayesian (FB) approach is implemented using the Markov Chain Monte Carlo (MCMC) method with Gibbs sampling in the MCMC procedure for SAS 9.4. The fully conditional distributions necessary to implement a Gibbs sampler are presented in the Supplementary Material (file FCD.docx). As for the EB approaches described above, we used the GAMINV function for the Gamma and inverse Gamma distributions. We used an inverse-Wishart prior distribution, W^−1^(*v*;Ω), for the variance–covariance matrix $\varphi$ of the log-variance components, where ν is the degrees of freedom equal to the number of variance components, and *Ω* is a scale matrix as diagonal matrix with values 1 (Schuurman et al. 2016). The hyper-parameters *α* and *β* in the Gamma or inverse Gamma prior distributions were estimated using the maximum likelihood (ML) methods (Bar and Schifano 2011). The log-likelihood function for these two parameters was described in Lonnstedt and Britton (2005). For the fixed effect of cultivars, a normal prior distribution was specified with zero mean and a variance equal 10^10^. We ran this estimation method for 10,000 iterations with a burn-in phase of 1000 iterations and with a thinning interval of 10. To evaluate MCMC convergence, we used Gelman–Rubin test (Gelman and Rubin 1992). The summary statistics of posterior distributions and results of Gelman–Rubin test for the FB approach for different prior distributions of variance components are presented in the Supplementary Material (Table S1). In Table S2 in the Supplementary Material, the results of the sensitivity analysis are presented.

**Fig. 2:**
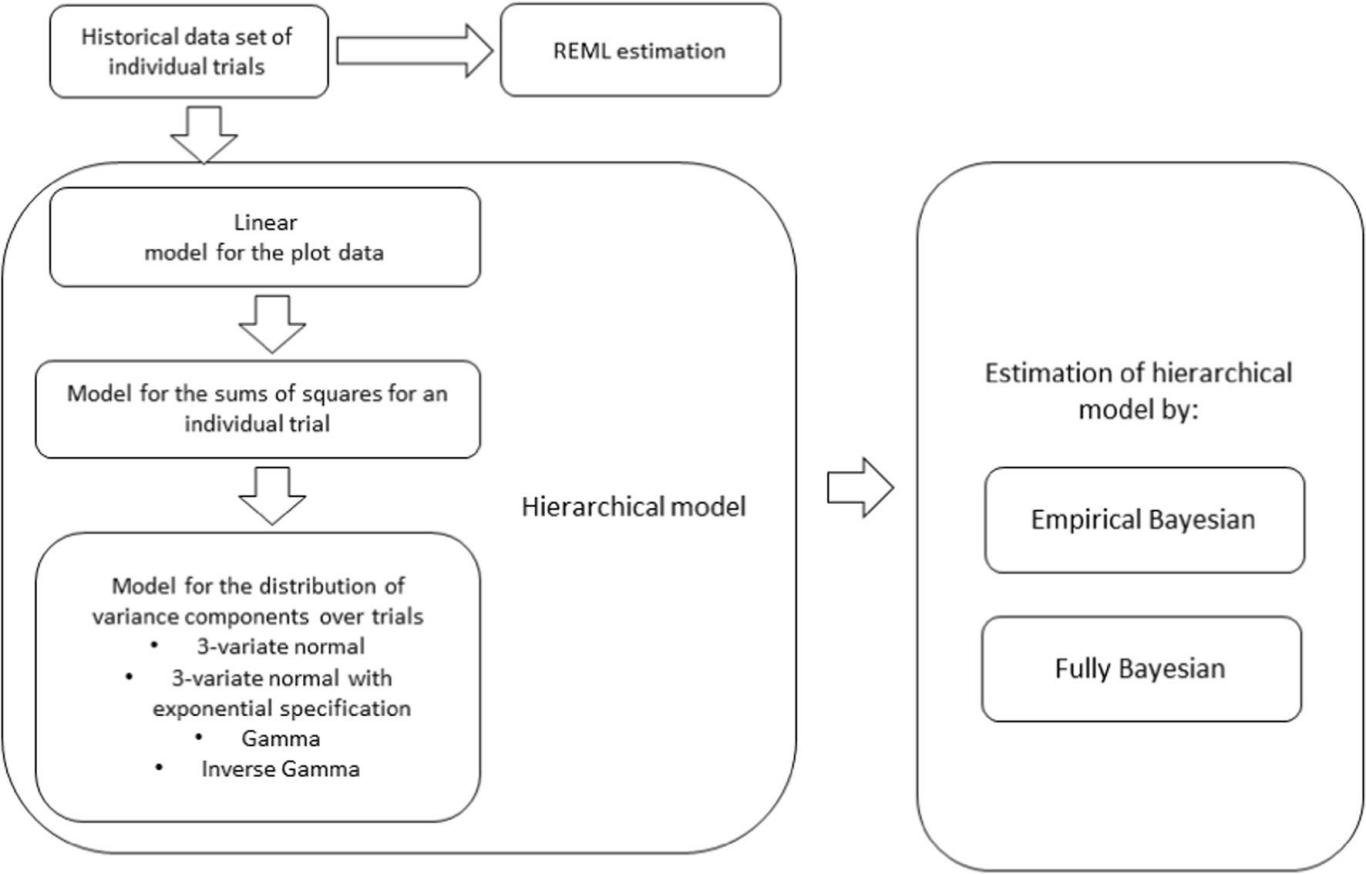
Diagram showing the main stages carried out during these studies

To sum up, we presented a hierarchical model for estimating variance components from individual trials, which consists of the following stages. In the first stage of estimation, we used a linear mixed model for the plot data, deriving the expected ANOVA sums of squares for an individual trial. The conditional distributions of the sums of squares are scaled Chi-squared distributions. Four different models were used for the distribution of variance components over trials. In order to summarise the performed analyses and their succession, Fig. 2 shows the main stages of the analyses.

### Simulations

The motivation for this study was to assess if strength can be borrowed across trials in estimating within-trial variance components. This is expected to be particularly worthwhile for the block variance in case of a limited number of blocks and small block size, in which case REML estimates frequently converge to zero (Verdooren 1982), thus effectively reducing the model by dropping the block effect and foregoing any within-replicate adjustments. Our rationale here is that incomplete blocks are always expected to capture some of the within-replicate heterogeneity, and hence, an estimate of exactly zero is not usually plausible. A similar reasoning applies to the variance for replicates, even though there is not usually any inter-replicate information to be recovered. This reasoning leads to our expectation that a Bayesian approach can improve the mixed model analysis of individual trials. Our suggested approach is to simply plug in the variance component estimates coming out of our hierarchical models into the mixed model package, which is then used to solve the mixed model equations. The purpose of the simulation is to test our hypothesis that the Bayesian approaches can improve the recovery of inter-block information and hence lead to more accurate estimates of treatment means and their differences. The simulation study is based on the historical data set from the Polish Post-Registration Variety Testing System. On this basis, we simulated individual trials (*n* = 1000). We used two types of simulated data sets, one with a small number of blocks (2) and small size of blocks (3 plots per block), and one with a relatively large number (10 blocks) and size of blocks (10 plots per block). In total, we had six varieties for the first simulated set of individual trials and 100 varieties for the second type. Both types of simulated individual trials had two repetitions. Randomisations in simulated individual trials were carried out using the optimisation built into the OPTEX procedure in the SAS package (Piepho 2015). We are aware that the scenario with six cultivars is rarely encountered in practice. Our main reason for including this scenario is that it presents a significant likelihood for the problem of zero variance components to occur, thereby allowing for rigorous testing of the proposed methods as well as the standard practices.

Our approach to simulation is based on estimating variance components using sums of squares based on a historical data set. Grain yield values were simulated using variance components generated from the fitted hierarchical model on the basis of sum of squares (from Table 1). Using these simulated variance components, the individual random effects in model (1) were simulated using a pseudo-random number generator based on the fitted Bayesian hierarchical model. For each simulated individual trial, we determined the mean squared error of estimated treatment (cultivar) differences (MSED) for all possible treatment means (cultivar means) pairwise comparisons. This was averaged for all simulated trials separately for each model. The lower the value of the MSED, the more efficient the recovery inter-block information (Möhring et al. 2015). Additionally, for actual variances for new individual trials, Bayesian estimates based on the “known” prior were used to evaluate the prediction of estimated variance components using the mean squared error (MSE).

## Results

### Historical data set

**Fig. 3:**
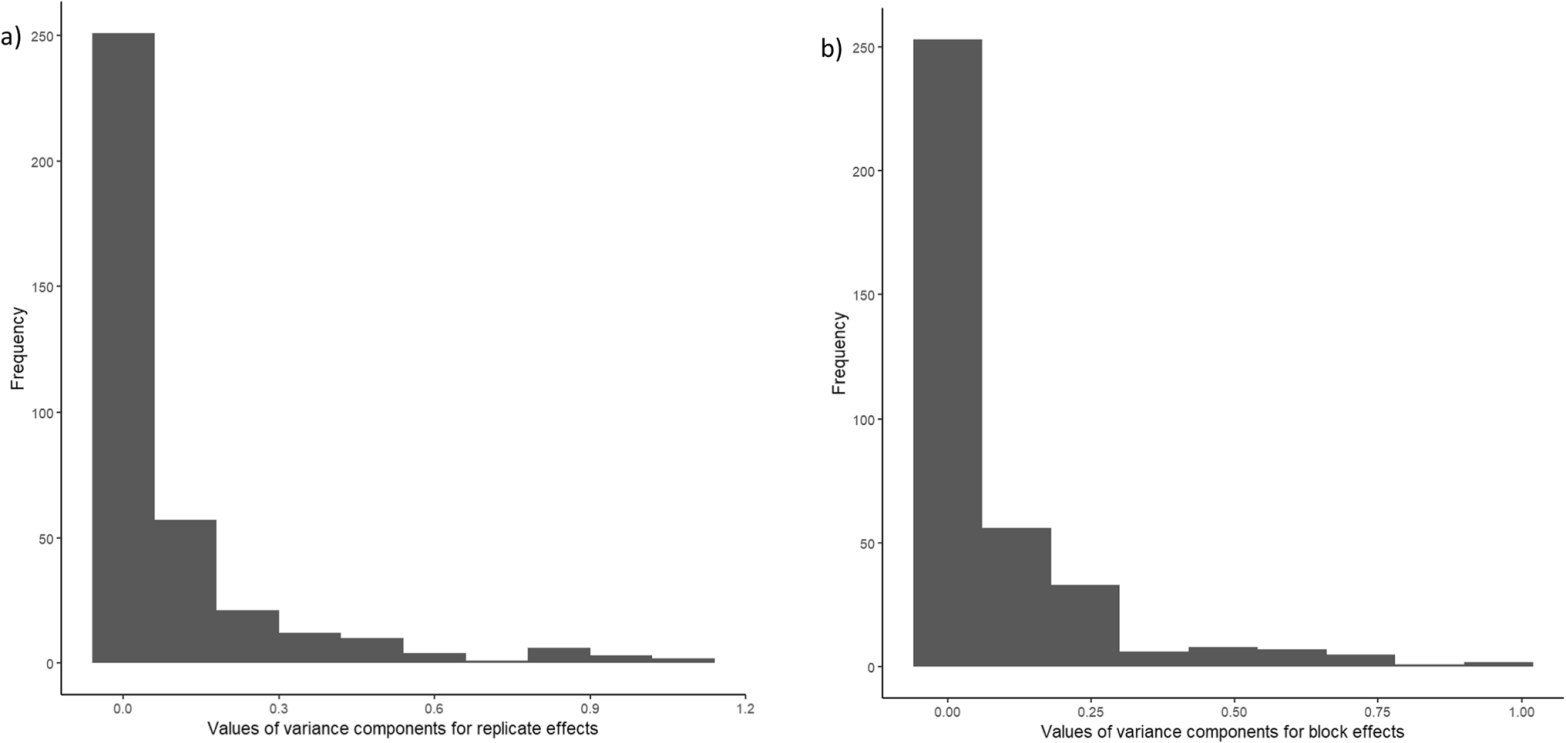
Values of variance components for replicate effects (**a**) and block effects (**b**) across individual trials from historical data set estimated by REML model

Figure 3 shows the distribution of REML estimates of the variance components for the replicate, block and error effects across all studies. Especially for the replicate and block effect, we observed a relatively large proportion of zero values for the estimated variance components. For the replicate variance, zero values were observed in 24.45% of the individual trials, and for the block variance, zero values were observed in 27.5% of the individual trials.

### Simulation

Table 2 presents the values of the Akaike Information Criterion (AIC), which measures the goodness of model fit. The highest AIC values were observed for the REML estimation method. Lower values of this model fitting criterion were observed for FB models compared to EB models. However, among the FB models, the type of data influenced whether the models had Gamma or inverse Gamma parameterisation.

**Table 2:** The Akaike Information Criterion (AIC) for study models in historical data set and in two simulated data sets

Estimation methods	Averaged of AIC for study data sets		
	Historical data set	3 plots per block	10 plots per block
REML	681.38	331.77	578.28
3-variate normal	610.42	231.13	494.74
3-variate normal with EB exponential specification	612.87	235.71	494.65
Gamma	603.84	224.12	487.32
Inverse Gamma	602.33	222.81	486.93
3-variate normal	604.68	214.27	486.84
3-variate normal with FB exponential specification	604.11	213.93	486.13
Gamma	597.92	202.73	473.28
Inverse Gamma	597.86	203.45	472.11

The mean squared error of estimated treatment differences (MSED) are presented in Table 3. In general, we observe a smaller variation in the value of this statistic for the scenario with a large number of cultivars in trials (10 plots per block), the differences between the REML method and any of Bayesian approaches are really small. In contrast, the scheme with a relatively small number of cultivars (three plots per block), the difference in MSED values between the REML method and any Bayesian approaches was higher than for 10 plots per block. For the simulation scheme with three plots per block and application of the EB approach, the lowest MSED value was observed for the model with inverse Gamma distribution of estimated variance components, while for the FB approaches, the lowest MSED value was observed for the model with Gamma distribution. On the other hand, for 10 plots per block, we observed the reverse reaction for MSED values. For the EB approach, the lowest MSED value was observed for the Gamma model, and for the Bayesian approaches, the lowest MSED was found for the inverse Gamma model.

**Table 3:** The mean squared error of estimated treatment differences (MSED) for study models in two simulated data sets

Estimation methods	Averaged of MSED for study data sets	
	3 plots per block	10 plots per block
REML	2.6126	2.0755
3-variate normal	1.9195	1.9965
3-variate normal with EB exponential specification	1.9181	1.9443
Gamma	1.9099	1.9221
Inverse Gamma	1.8919	1.9081
3-variate normal	1.8543	1.9906
3-variate normal with FB exponential specification	1.8291	1.8996
Gamma	1.8011	1.9034
Inverse Gamma	1.8102	1.8855

*EB* Empirical Bayes approach, *FB* fully Bayesian approach

The second parameter used for evaluating the proposed models was the mean squared error (MSE) for the individual variance components, whose averaged values are presented in Table 4. In general, regardless of the effect type, we observed slightly higher average MSE for the simulation scheme with a small number of cultivars and small blocks size than with a large number of cultivars and a large block size. The highest values of this parameter, regardless of the type of the variance component, were observed for the model where the parameters were estimated using the REML method. On the other hand, among the models using both approaches, the smallest differences in MSE values were observed for variance components for error. Regarding the variance components for replication and block, the lowest values of MSE in both simulation schemes were observed for the inverse Gamma distribution with both schemes of simulated data with the ML approach and in the FB approaches. For these two types of variance components, slightly lower mean MSE values were observed for the Bayesian approaches than for the ML approach.

**Table 4:** The mean squared error (MSE) of variance components for study models in two simulated data sets

Estimations	Averaged of MSE for study data sets					
	Variance components for replication		Variance components for block		Variance components for error	
	3 plots per block	10 plots per block	3 plots per block	10 plots per block	3 plots per block	10 plots per block
REML	0.4726	0.2545	0.3953	0.1229	0.0369	0.0131
3-variate normal	0.3696	0.2298	0.3133	0.1109	0.0321	0.0123
3-variate normal with EB exponential specification	0.3687	0.2292	0.3128	0.1099	0.0321	0.0121
Gamma	0.3483	0.2291	0.3051	0.1091	0.0320	0.0119
Inverse Gamma	0.3466	0.2284	0.3048	0.1084	0.0320	0.0121
3-variate normal	0.3511	0.2265	0.3088	0.1017	0.0317	0.0109
3-variate normal with FB exponential specification	0.3502	0.2261	0.3081	0.1012	0.0323	0.0111
Gamma	0.3398	0.226	0.3029	0.1019	0.0321	0.0109
Inverse Gamma	0.3387	0.2244	0.3022	0.1006	0.0318	0.0103

*EB* Empirical Bayesian approach, *FB* fully Bayesian approach

## Discussion

Our simulation approach assumes that spatial trend in a field trial can be captured by positive variance components for both replicates and blocks. Furthermore, our hierarchical models impose the constraint that all variance component estimates must be positive. Two issues may potentially be raised in this context. (i) Instead of using a randomisation-based model for simulating the data, which ensures that the underlying block variances are truly positive, one could consider alternative models and methods that simulate more realistic irregular field trends and spatial correlations. Moreover, uniformity trial data could be used, superimposing treatment effects to simulate realistic variety trial data. A substantial number of uniformity trials would be needed to obtain a solid basis for simulation. These simulation options could replicate the presence of negative covariance among observations within the same block. Such patterns may have a real basis, as pointed out, e.g. by Nelder (1954, 1977), Hocking (1985) and Welham et al. (2014). While our simulation-based approach precludes such negative correlations in the data-generating mechanism, it does allow for realisations of plot values that do have this feature. Furthermore, our randomisation-based simulation approach favours neither of our approaches considered for analysis, because all assume the same trial-level linear mixed model for analysis. (ii) When treatments and block factors are orthogonal, such as in a randomised complete block design or a split plot design, the exact F-test for treatment effects of interest is a ratio of mean squares based on ordinary least squares. If a mixed model package is used, these F-statistics will be reproduced when allowing REML to produce negative variance components. This may lead to the conclusion that the non-negativity constraint usually imposed by mixed model packages should generally be lifted. While this usually reproduces exact F-statistics in the simple balanced cases alluded to above, it does have the problem of occasionally causing convergence problems (Frey et al. 2024). When the design involves incomplete blocks, ordinary least square is no longer a preferable option. Instead, generalised least squares are used using estimates of the variance components for blocks. Furthermore, lifting the non-negativity constraint in this case would exacerbate convergence problems with REML. It may be added that in the case of variety trials, significance tests are of less relevance than point estimates of treatment effects. The problem with the default setting of REML packages is that terms whose variance converges to zero are dropped from the model. However, randomisation theory dictates that the experimental design of field trials should be included in the statistical model for valid standard errors to be estimated for genotype (treatment) effects (Nelder 1965a, b; Bailey 2008). Our approach to impose positivity of all variance components ensures that no strata are inadvertently dropped from the model, which would compromise standard errors for some treatment comparisons. All of these considerations enforce our approach to require positive variance components for resolvable incomplete block design.

Our results show that the use of a Bayesian framework with empirically informed priors increases the prediction accuracy of variation component estimates for replications, blocks and error, and makes the recovery of inter-block information more effective in individual trials carried out according to an alpha design. This contributes to an increase in the effectiveness of the cultivar assessment and thereby allows plant breeders to make better selections and farmers to make a more reliable choices of cultivars. The Bayesian paradigm is gaining popularity in many areas of research, including crop improvement. It appears to us that the main reason for this gained popularity is the availability of computational resources and powerful software (McCarthy 2007; Green et al. 2020). With complex models that are difficult to implement with frequentist methods, Bayesian methods may offer the additional advantage of computational convenience. Most applications use non-informative priors, however, and where informative priors are used, there is often only a limited objective basis for their choice (Hobbs and Hooten 2015; Lemoine 2019).

This paper has demonstrated that the use of such methods promises non-negligible gains in precision compared to the REML method, gains that come essentially for free once the approach is implemented. The differences in accuracy and efficiency in the recovery inter-block information between the evaluated estimation methods and used informative prior distribution specifications were slight. However, a small advantage in efficiency was observed for the FB approach with Gamma or inverse Gamma prior distributions. Consequently, it can be concluded that the use of informative priors improved the efficiency in the recovery inter-block information and prediction accuracy of Bayesian methods in individual trial analyses for a two-stage approach. However, research by Polson and Scott (2012) and by Gelman (2006) indicates that the half-Cauchy distribution for variance components may be a viable alternative. Our focus here has been on alpha designs, mainly because of their great popularity, but our approach is readily applicable with other blocked experimental designs, such as non-resolvable or resolvable row–column designs (John and Williams 1995).

Plant breeding programmes and official variety testing systems typically have substantial amounts of historical data on past trials at their disposal (Smith et al. 2006; Laidig et al. 2017). The results of previously conducted analyses, along with the mean values and variability of the variance components of errors or variance components for other effects specific to a given experimental design, can help determine a priori distributions and increase the efficiency of individual trial analyses. For each of the considered response variables (yield or grain quality traits), separate prior distribution specifications should be determined.

In this paper, to build a prior distribution, we used a historical data set from a relatively long period of ten growing seasons. De Silva et al. (2013) and Azevedo et al. (2022) suggest that the period for which the informative priori is built should be as short as possible, they even recommend that it relates to the last period of field trials and does not recommend building priors based on research results from many years. However, their research relates to the estimation and evaluation of prediction accuracy for various types of genetic parameters. The limitation of the number of growing seasons used to determine the prior distribution may be justified by the fact that there is a fairly large rotation of genotypes in trials from year to year, and thus the occurrence of genetic and breeding progress. In our case, the parameters we use, i.e. the variance components for replicates, blocks and error, are strongly related to specific trial location and year. Thus, it is important to have a large number of trials from historical sources to build priors.

Our comparative assessment of the Bayesian (empirical and fully) framework and the REML method was conducted for two specific numbers of blocks per replicate and numbers of plots per block. In real data sets, the number of blocks and plots in blocks is often differentiated between trials, and our historical one can be considered a representative sample. It should be stressed, however, that the numbers of blocks and plots in blocks were not strongly differentiated in our case, so this simplification does not affect the assessment and conclusion about the usefulness of the Bayesian methods.

Given that the Bayesian framework is ideally suited to exploit the empirically available information to inform priors for variance components needed in the analysis of individual trials, it is quite surprising that approaches such as the one suggested here do not seem to be in common usage. There may be several reasons for this. One is that there is a lack of comparative research on the effectiveness of this approach in the analysis of individual trials. More research would contribute to the dissemination of these methods. Another reason is that Bayesian methods may be considered complicated and difficult to apply by plant breeders and other researchers involved in the evaluation of varieties.

The use of the Bayesian approaches allowed the individual trial analysis to be more efficient than by the REML method used in the first stage of the two-stage approach to MET analysis. The Bayesian framework suggested here can also be considered for the variance components pertaining to random effects for genotypes, environments and genotype–environment interaction in the second stage, and this opportunity also deserves consideration in future work. The main challenge here will be the assembly of large historical data bases, allowing accurate estimation of priors. The challenge is increased when environments are partitioned by years and locations, giving rise to a three-way linear model with factors years, locations and genotypes. Part of the challenge is to delineate subsets of years in a long-term data set from which to estimate all variance components or obtain all required sums of squares. Perhaps, the most natural partition in registration trials is according to series of trials testing the same set of genotypes. In official variety testing, each year a new set of genotypes enters the tests, and this set is then tested for 2 or 3 years. Analysing the MET for these sets separately should provide a good basis for informing the priors.

Potentially, in the analysis of METs, a single-stage Bayesian hierarchical modelling approach could be used to improve the recovery of inter-block information, which may be an alternative to the two-stage analysis used so far. However, we see practical challenges in applying this approach to the MET analysis. We have lots of trials, and the sets of genotypes in these historical data are very variable and partly even disconnected. So there is little if any inter-trial information on the comparisons of interest in a new trial. The prior information on the between-trial variance components (environmental main effects and genotype–environment interaction) is usually scant. In fact, any long-term data available from a single variety testing programme would yield only a single variance component estimate for any such between-trial variance component (Hu and Spilke 2011). This provides only a weak basis for objectively informing a prior on between-trial variance components. To make progress, it would be useful to have multiple MET data sets that allow building an objectively informed prior for between-trial variance components.

### Electronic supplementary material

Below is the link to the electronic supplementary material.

Supplementary file1 (DOCX 20 KB)

Supplementary file2 (DOCX 63 KB)

## Data Availability

The data sets generated during and/or analysed during the current study are available from the corresponding author on reasonable request.

## Appendix

Alpha designs have the property of orthogonal block structure and general balance (Nelder 1965a, b; Houtman and Speed 1983)). The conditional independence of sums of squares SS_*r*_, SS_*b*_ and SS_*e*_ can be established using this property.

(a) An analysis of variance can be computed under the usual null model of no treatment effects, assuming joint normality of all the observations (Nelder 1965a). Denote the sums of squares for replicates, blocks and plots under the null model as SS*(Reps)*, SS*(Blocks)* and SS*(Plots)*, respectively. Due to the general balance property of alpha designs, leading to orthogonality of the variance–covariance structure, these sums of squares are conditionally independent. Note that due to the orthogonality of replicates and treatments under the full model, SS(*Reps*) coincides with SS_*r*_ in our analysis.

(b) Under the full model including treatment effects, the block and plot strata contain information on treatment contrasts. Thus, in the general analysis of variance (Nelder 1965b), SS*(Blocks)* is decomposed as.

SS(*Blocks*) = SS(*Treat*)_*b*_ + SS(*Residual*)_*b*_,

where the subscript b indicates the block stratum, and the two components SS(*Treat*)_*b*_ and SS(*Residual*)_*b*_ are independent. Observe here that SS(*Residual*)_*b*_ is identical to SS_*b*_ in our analysis. The sum of squares SS(*Plots*) can be similarly decomposed as.

SS(*Plots*) = SS(*Treat*)_*p*_ + SS(*Residual*)_*p*_,

where the subscript p indicates the plot stratum, and the two components SS(*Treat*)_*p*_ and SS(*Residual*)_*p*_ are independent. Observe here that SS(*Residual*)_*p*_ is identical to SS_*e*_ in our analysis.

It follows from all of this that SS_*r*_, SS_*b*_ and SS_*e*_ are indeed conditionally independent.
