# Supplementary material for: Hierarchical modelling of variance components makes analysis of resolvable incomplete block designs more efficient
Source: Theor Appl Genet. 2024 May 16;137(6):134. doi: 10.1007/s00122-024-04639-4 (PMC11098934; doi:10.1007/s00122-024-04639-4)
Supplement: Supplementary file 1 — Supplementary file1 (DOCX 20 KB) [file 122_2024_4639_MOESM1_ESM.docx]

Supplementary materials

**Hierarchical modelling of variance components makes analysis of resolvable incomplete block designs more efficient**

Marcin Studnicki, Hans Peter Piepho

The full conditional distributions used for Gibbs sampling in SAS MCMC procedure is defined as follows (Gelman et al., 2013):

For given variance components, $\sigma_{i}=\left( \sigma_{r}^{2},\sigma_{b}^{2},\sigma_{e}^{2} \right)^{T}$, the sums of squares, ${SS}_{i}$, have independent scaled central chi-squared distributions:

$\frac{SS_{i}|\sigma_{i}}{E\left( MS_{i} \right)}\sim\chi_{v_{i}}^{2}$

For $\sigma_{i}$ assumed prior

$\sigma_{i}=\left( \begin{matrix} \log\left( \sigma_{r}^{2} \right) \\ \log\left( \sigma_{b}^{2} \right) \\ \log\left( \sigma_{e}^{2} \right) \end{matrix} \right)\sim MVN\left[ \theta=\left( \begin{matrix} \theta_{r} \\ \theta_{b} \\ \theta_{e} \end{matrix} \right),\Sigma=\left( \begin{matrix} \varphi_{r}^{2} & \varphi_{rb} & \varphi_{re} \\ & \varphi_{b}^{2} & \varphi_{be} \\ & & \varphi_{e}^{2} \end{matrix} \right) \right]$

where MVN[] denotes a multivariate normal distribution.

The 3-variate normal distribution has priors:

$\theta\sim MVN(\mu,\Omega)$

where $\mu$ is vector of means and $\Omega$ is variance-covariance matrix. The values for $\mu$ and $\Omega$ were obtain used historical data set,

and

$$\Sigma\sim IW(v,S)$$

where *IW*() denotes inverse-Wishart distribution , ν is the degrees of freedom ν > n-1, where (n, n) is the dimension of ∑ and $S$ is a scale matrix which is a (n, n) deterministic symmetric positive definite matrix. The larger the df , the more informative is the distribution (Gelman et al., 2013), we have chosen v = 10. The specification of S scale matrix is based on estimates of the variances of the random effects from historical data set. This approaches to formed of S scale matrix from data were show by Schuurman (2016) and Zhang (2021).

The joint posterior distribution has the form:

$$p\left( \sigma_{i}, \theta, \Sigma\right|{SS}_{i})\propto\frac{1}{{{SS}_{i}}^{\frac{n}{2}+1}}exp\left\{ \frac{1}{2{SS}_{i}}(\sigma_{i}-\left( \theta\right)_{i}-\det\left( \Sigma\right))^{'}\Sigma^{-1}{\sum_{i=1}^{n} ({SS}_{i}-\left( \theta\right)_{i})} \right\}\times$$

$$\prod_{i=1}^{n} \left[ \frac{1}{{\sigma_{i}}^{\frac{n}{2}+1}}exp\left\{ -{SS}_{i}-\frac{1}{\sigma_{i}}\det\left( \Sigma\right)^{-1} \right\} \right]$$

1. **The 3-variate normal and 3-variate normal with exponential parameterization for the variance components**

The fully conditional distributions for $\sigma_{i}$:

$$p\left( \sigma_{i} \right|{SS}_{i},\theta, \Sigma)\propto det(\Sigma)^{-(n+1)/2}\times exp\left\{ -tr\frac{det(\Sigma^{-1})}{2} \right\}$$

$$\times det(\Sigma)^{-n/2}exp\left\{ -tr\frac{\left( \theta\right)_{i}\Sigma^{-1}}{2} \right\}$$

$$\propto\sum_{i=1}^{n} det(\Sigma)^{-(n+1)/2}\times exp\left\{ -tr(\left( {SS}_{i}+\left( \theta\right)_{i} \right)\Sigma^{-1})/2 \right\}$$

1. **The gamma distribution parameterizations**

The fully conditional distributions for $\sigma_{i}$ is in the scaled by gamma distribution form:

$$p\left( \sigma_{i} \right|{SS}_{i},\theta, \Sigma,)\propto\sum_{i=1}^{kn} exp({\varphi_{k}}^{-a_{i}+n+2})exp\left\{ -\sum_{i=1}^{n} SSi\frac{{{a_{i}b}_{i}}}{2} \right\}$$

$$\times\prod_{i=1}^{n} {\sigma_{i}}^{-n/n}exp\left\{ -\sum_{i=1}^{n} SSi\sum_{i=1}^{n} \frac{{{a_{i}b}_{i}}}{2} \right\}$$

$$\propto{\prod_{i=1}^{n} {\sigma_{i}}^{-\frac{-(a_{i}+n)}{2}-1}}exp\left\{ -\sum_{i=1}^{n} {(a}_{i}b_{i})/2 \right\}$$

1. **The inverse gamma distribution parameterizations**

The fully conditional distributions for $\sigma_{i}$ is in the scaled by inverse gamma distribution form:

$$p\left( \sigma_{i} \right|{SS}_{i},\theta, \Sigma,)\propto\sum_{i=1}^{kn} exp({\varphi_{k}}^{-{a_{i}}^{-1}+n+2})exp\left\{ -\sum_{i=1}^{n} SSi\frac{{{a_{i}b}_{i}}^{-1}}{2} \right\}$$

$$\times\prod_{i=1}^{n} {\sigma_{i}}^{-n/n}exp\left\{ -\sum_{i=1}^{n} SSi\sum_{i=1}^{n} \frac{{{a_{i}b}_{i}}^{-1}}{2} \right\}$$

$$\propto{\prod_{i=1}^{n} {\sigma_{i}}^{-\frac{-({a_{i}}^{-1}+n)}{2}-1}}exp\left\{ -\sum_{i=1}^{n} {(a}_{i}b_{i})^{-1}/2 \right\}$$

Reference

Gelman, A., Carlin, J.B., Stern, H.S., Dunson, D.B., Vehtari, A.,& Rubin, D.B. (2013).Bayesian data analysis (3d ed.).BocaRaton, FL: Chapman & Hall/CRC

Schuurman NK, Grasman RPPP, Hamaker EL (2016) A comparison of inverse-wishart prior specifications for covariance matrices in multilevel autoregressive models. Multivar Behav Res 51:185–206. https://doi.org/10.1080/00273171.2015.1065398

Zhang, Z. (2021). A Note on Wishart and Inverse Wishart Priors for Covariance Matrix. Journal of Behavioral Data Science, 1(2), 119-126. https://doi.org/10.35566/jbds/v1n2/p2
