# Supplementary material for: Hierarchical modelling of variance components makes analysis of resolvable incomplete block designs more efficient
Source: Theor Appl Genet. 2024 May 16;137(6):134. doi: 10.1007/s00122-024-04639-4 (PMC11098934; doi:10.1007/s00122-024-04639-4)
Supplement: Supplementary file 2 — Supplementary file2 (DOCX 63 KB) [file 122_2024_4639_MOESM2_ESM.docx]

Supplementary materials

Table S1. Summary statistics of posterior distributions for Full Bayesian approach

| Parameters | 3-variate normal | | | | | | | 3-variate normal with exponential parameterization for variance components | | | | | | |
| --- | --- | --- | --- | --- | --- | --- | --- | --- | --- | --- | --- | --- | --- | --- |
|  | Mean | SE | Credible Intervals | | Highest Posterior Density | | Gelman-Rubin convergence diagnostic Rc | Mean | SE | Credible Intervals | | Highest Posterior Density | | Gelman-Rubin convergence diagnostic Rc |
|  |  |  | Lower | Upper | Lower | Upper |  |  |  | Lower | Upper | Lower | Upper |  |
| θr | 5.79 | 0.36 | 5.11 | 6.48 | 5.03 | 6.38 | 1.0022 | 5.98 | 0.47 | 5.24 | 6.74 | 5.16 | 6.64 | 1.0018 |
| θb | 3.86 | 0.18 | 3.54 | 4.24 | 3.49 | 4.18 | 1.0102 | 4 | 0.39 | 3.64 | 4.44 | 3.59 | 4.38 | 1.0094 |
| θe | 0.66 | 0.06 | 0.2 | 0.8 | 0.20 | 0.79 | 1.0002 | 0.96 | 0.24 | 0.68 | 1.28 | 0.67 | 1.26 | 1.0019 |
| $\varphi_{r}^{2}$ | 2.06 | 0.17 | 0.82 | 3.68 | 0.81 | 3.62 | 1.0029 | 2.2 | 0.43 | 1.07 | 3.71 | 1.05 | 3.65 | 1.0034 |
| $\varphi_{b}^{2}$ | 0.34 | 0.2 | 0.27 | 0.55 | 0.27 | 0.54 | 1.0352 | 0.47 | 0.4 | 0.36 | 0.67 | 0.35 | 0.66 | 1.0283 |
| $\varphi_{e}^{2}$ | 0.25 | 0.08 | 0.08 | 0.38 | 0.08 | 0.38 | 1.0291 | 0.46 | 0.31 | 0.24 | 0.71 | 0.24 | 0.70 | 1.0304 |
| $\varphi_{rr}$ | 6.83 | 1.65 | 4.23 | 8.76 | 4.18 | 8.66 | 1.0037 | 7.11 | 1.85 | 3.96 | 11.32 | 3.91 | 11.19 | 1.0138 |
| $\varphi_{re}$ | 8.13 | 2.56 | 5.74 | 10.82 | 5.66 | 10.66 | 1.0173 | 8.25 | 2.74 | 5.75 | 11.11 | 5.67 | 10.95 | 1.019 |
| $\varphi_{be}$ | 13.82 | 0.47 | 11.65 | 16.06 | 11.42 | 15.74 | 1.0208 | 14.04 | 0.59 | 11.99 | 16.28 | 11.75 | 15.96 | 1.0214 |
|  | Gamma | | | | | | | Invers gamma | | | | | | |
|  | Mean | SE | Credible Intervals | | Highest Posterior Density | | Gelman-Rubin convergence diagnostic Rc | Mean | SE | Credible Intervals | | Highest Posterior Density | | Gelman-Rubin convergence diagnostic Rc |
|  |  |  | Lower | Upper | Lower | Upper |  |  |  | Lower | Upper | Lower | Upper |  |
| αr | 1.22 | 0.47 | 0.93 | 2.43 | 0.92 | 2.39 | 1.0281 | 1.21 | 0.43 | 0.97 | 2.47 | 0.96 | 2.43 | 1.0192 |
| αb | 0.28 | 0.04 | 0.12 | 0.62 | 0.12 | 0.61 | 1.0032 | 0.3 | 0.03 | 0.14 | 0.61 | 0.14 | 0.60 | 1.0056 |
| αe | 4.99 | 0.45 | 3.79 | 5.92 | 3.75 | 5.86 | 1.0184 | 5.02 | 0.33 | 3.82 | 5.82 | 3.78 | 5.76 | 1.0192 |
| βr | 8.36 | 1.22 | 5.63 | 10.91 | 5.57 | 10.79 | 1.0049 | 8.22 | 0.99 | 5.25 | 9.85 | 5.19 | 9.74 | 1.0056 |
| βb | 3.81 | 0.38 | 2.65 | 4.76 | 2.60 | 4.67 | 1.0261 | 3.44 | 0.41 | 2.43 | 4.02 | 2.38 | 3.94 | 1.0241 |
| βe | 3.4 | 0.91 | 2.41 | 4.11 | 2.37 | 4.05 | 1.0282 | 3.25 | 1.08 | 2.36 | 4.19 | 2.32 | 4.13 | 1.0243 |
| $\varphi_{rr}$ | 6.99 | 1.67 | 3.44 | 9.83 | 3.39 | 9.68 | 1.0119 | 6.98 | 1.61 | 3.39 | 9.97 | 3.34 | 9.82 | 1.0105 |
| $\varphi_{re}$ | 8.28 | 2.71 | 5.68 | 10.83 | 5.62 | 10.72 | 1.0108 | 8.19 | 2.66 | 5.67 | 10.78 | 5.61 | 10.67 | 1.0094 |
| $\varphi_{be}$ | 13.99 | 0.6 | 11.89 | 15.48 | 11.72 | 15.26 | 1.0082 | 13.96 | 0.52 | 11.86 | 15.23 | 11.69 | 15.02 | 1.0063 |

Table S2. Sensitive analysis of posterior distributions for Full Bayesian approach

| Parameters | Weights for hyperparameters | 3-variate normal | | | 3-variate normal with exponential parameterization for variance components | | |
| --- | --- | --- | --- | --- | --- | --- | --- |
|  |  | Mean | 95% Credible Intervals | | Mean | 95% Credible Intervals | |
|  |  |  | Lower | Upper |  | Lower | Upper |
| θr | 0.8 | 5.792984 | 5.112634 | 6.48334 | 5.983082 | 5.242701 | 6.743474 |
|  | 1 | 5.79554 | 5.114889 | 6.4862 | 5.985722 | 5.245014 | 6.746449 |
|  | 1.2 | 5.805013 | 5.12325 | 6.496803 | 5.995506 | 5.253587 | 6.757477 |
| θb | 0.8 | 3.861989 | 3.541824 | 4.242185 | 4.002061 | 3.641876 | 4.442288 |
|  | 1 | 3.863693 | 3.543387 | 4.244057 | 4.003827 | 3.643483 | 4.444248 |
|  | 1.2 | 3.870009 | 3.549179 | 4.250994 | 4.010372 | 3.649439 | 4.451513 |
| θe | 0.8 | 0.66034 | 0.200103 | 0.800412 | 0.960495 | 0.68035 | 1.28066 |
|  | 1 | 0.660631 | 0.200191 | 0.800765 | 0.960919 | 0.680651 | 1.281225 |
|  | 1.2 | 0.661711 | 0.200519 | 0.802074 | 0.962489 | 0.681763 | 1.283319 |
| $\varphi_{r}^{2}$ | 0.8 | 2.061062 | 0.820423 | 3.681897 | 2.201134 | 1.070551 | 3.711912 |
|  | 1 | 2.061971 | 0.820785 | 3.683521 | 2.202105 | 1.071024 | 3.71355 |
|  | 1.2 | 2.065342 | 0.822126 | 3.689542 | 2.205705 | 1.072775 | 3.71962 |
| $\varphi_{b}^{2}$ | 0.8 | 0.340175 | 0.270139 | 0.550283 | 0.470242 | 0.360186 | 0.670345 |
|  | 1 | 0.340325 | 0.270258 | 0.550526 | 0.47045 | 0.360344 | 0.670641 |
|  | 1.2 | 0.340882 | 0.2707 | 0.551426 | 0.471219 | 0.360933 | 0.671737 |
| $\varphi_{e}^{2}$ | 0.8 | 0.250129 | 0.080041 | 0.380196 | 0.460237 | 0.240124 | 0.710366 |
|  | 1 | 0.250239 | 0.080077 | 0.380364 | 0.46044 | 0.24023 | 0.710679 |
|  | 1.2 | 0.250648 | 0.080207 | 0.380985 | 0.461193 | 0.240622 | 0.711841 |
| $\varphi_{rr}$ | 0.8 | 6.83352 | 4.23218 | 8.764515 | 7.113664 | 3.962041 | 11.32583 |
|  | 1 | 6.836535 | 4.234047 | 8.768382 | 7.116803 | 3.963789 | 11.33083 |
|  | 1.2 | 6.84771 | 4.240968 | 8.782715 | 7.128436 | 3.970268 | 11.34935 |
| $\varphi_{re}$ | 0.8 | 8.13419 | 5.742958 | 10.82558 | 8.254252 | 5.752963 | 11.11573 |
|  | 1 | 8.137779 | 5.745492 | 10.83035 | 8.257894 | 5.755502 | 11.12063 |
|  | 1.2 | 8.151081 | 5.754884 | 10.84806 | 8.271392 | 5.76491 | 11.13881 |
| $\varphi_{be}$ | 0.8 | 13.82712 | 11.656 | 16.06828 | 14.04724 | 11.99618 | 16.28839 |
|  | 1 | 13.83322 | 11.66115 | 16.07537 | 14.05343 | 12.00147 | 16.29558 |
|  | 1.2 | 13.85584 | 11.68021 | 16.10164 | 14.07641 | 12.02109 | 16.32221 |
|  |  | Gamma | | | Invers gamma | | |
|  |  | Mean | 95% Credible Intervals | | Mean | 95% Credible Intervals | |
|  |  |  | Lower | Upper |  | Lower | Upper |
| αr | 0.9 | 1.220629 | 0.930479 | 2.431252 | 1.210624 | 0.9705 | 2.471273 |
|  | 1 | 1.221167 | 0.93089 | 2.432325 | 1.211158 | 0.970928 | 2.472363 |
|  | 1.1 | 1.223163 | 0.932411 | 2.436301 | 1.213138 | 0.972515 | 2.476405 |
| αb | 0.9 | 0.280144 | 0.120062 | 0.62032 | 0.300155 | 0.140072 | 0.610314 |
|  | 1 | 0.280268 | 0.120115 | 0.620593 | 0.300287 | 0.140134 | 0.610584 |
|  | 1.1 | 0.280726 | 0.120311 | 0.621608 | 0.300778 | 0.140363 | 0.611582 |
| αe | 0.9 | 4.992572 | 3.791953 | 5.923051 | 5.022587 | 3.821969 | 5.822999 |
|  | 1 | 4.994774 | 3.793626 | 5.925664 | 5.024803 | 3.823655 | 5.825569 |
|  | 1.1 | 5.002939 | 3.799827 | 5.935351 | 5.033017 | 3.829905 | 5.835091 |
| βr | 0.9 | 8.364308 | 5.632902 | 10.91562 | 8.224236 | 5.252706 | 9.855076 |
|  | 1 | 8.367999 | 5.635387 | 10.92044 | 8.227865 | 5.255023 | 9.859425 |
|  | 1.1 | 8.381677 | 5.644599 | 10.93829 | 8.241314 | 5.263613 | 9.875541 |
| βb | 0.9 | 3.811964 | 2.651366 | 4.762453 | 3.441773 | 2.431252 | 4.022072 |
|  | 1 | 3.813645 | 2.652536 | 4.764554 | 3.443291 | 2.432325 | 4.023846 |
|  | 1.1 | 3.819879 | 2.656871 | 4.772343 | 3.44892 | 2.436301 | 4.030424 |
| βe | 0.9 | 3.401752 | 2.411242 | 4.112118 | 3.251675 | 2.361216 | 4.192159 |
|  | 1 | 3.403253 | 2.412306 | 4.113932 | 3.25311 | 2.362258 | 4.194009 |
|  | 1.1 | 3.408816 | 2.416249 | 4.120657 | 3.258427 | 2.366119 | 4.200865 |
| $\varphi_{rr}$ | 0.9 | 6.993602 | 3.441773 | 9.835066 | 6.983597 | 3.391747 | 9.975138 |
|  | 1 | 6.996688 | 3.443291 | 9.839405 | 6.986678 | 3.393244 | 9.979539 |
|  | 1.1 | 7.008125 | 3.44892 | 9.855489 | 6.998099 | 3.39879 | 9.995852 |
| $\varphi_{re}$ | 0.9 | 8.284267 | 5.682927 | 10.83558 | 8.194221 | 5.672922 | 10.78556 |
|  | 1 | 8.287922 | 5.685435 | 10.84036 | 8.197836 | 5.675425 | 10.79031 |
|  | 1.1 | 8.30147 | 5.694728 | 10.85808 | 8.211237 | 5.684702 | 10.80795 |
| $\varphi_{be}$ | 0.9 | 13.99721 | 11.89613 | 15.48798 | 13.96719 | 11.86611 | 15.23785 |
|  | 1 | 14.00339 | 11.90138 | 15.49481 | 13.97336 | 11.87135 | 15.24457 |
|  | 1.1 | 14.02628 | 11.92083 | 15.52014 | 13.9962 | 11.89075 | 15.26949 |
